# *Keiferiaazapaensis* sp. nov., the first representative of the New World micromoth genus *Keiferia* Busck (Lepidoptera, Gelechiidae) associated with a member of Asteraceae

**DOI:** 10.3897/BDJ.13.e141827

**Published:** 2025-01-20

**Authors:** Héctor A. Vargas

**Affiliations:** 1 Universidad de Tarapacá, Facultad de Ciencias Agronómicas, Departamento de Recursos Ambientales, Arica, Chile Universidad de Tarapacá, Facultad de Ciencias Agronómicas, Departamento de Recursos Ambientales Arica Chile

**Keywords:** Atacama Desert, DNA barcoding, host plants, leaf miner larvae

## Abstract

**Background:**

The New World micromoth genus *Keiferia* Busck, 1939 (Lepidoptera, Gelechiidae, Gelechiinae, Gnorimoschemini) includes 21 described species, ten of which occur in South America. Like the tomato pinworm, *K.lycopersicella* (Walsingham, 1897), all the species of *Keiferia*, whose host plants have been documented, are associated exclusively with members of the family Solanaceae.

**New information:**

*Keiferiaazapaensis* sp. nov. is described and illustrated, based on adults reared from leaf miner larvae collected on the shrub *Trixiscacalioides* (Kunth) D. Don (Asteraceae) in the Atacama Desert, northern Chile. Despite this unusual host plant, a Maximum Likelihood analysis, based on mitochondrial DNA sequences, placed the new species within a well-supported *Keiferia* clade. The discovery of the trophic association between *K.azapaensis* sp. nov. and *T.cacalioides* represents the first record of a member of Asteraceae as a host plant for the micromoth genus *Keiferia*.

## Introduction

*Keiferia* Busck, 1939 (Lepidoptera, Gelechiidae, Gelechiinae, Gnorimoschemini) is a New World micromoth genus originally described to include four North American species whose male genitalia have a large, pointed, hook-shaped uncus, weak gnathos and apically enlarged valva (Busck 1939). The most recent world catalogue of the family Gelechiidae recognises 21 species in this genus, ten of which have been recorded in South America, only one of them being in Chile (Hobern et al. 2024). The best-known species is the tomato pinworm, *K.lycopersicella* (Walsingham, 1897), originally described from West Indies (Walsingham 1897); it is a pest of tomato crops whose larvae initially feed as leaf miners and, later, by tying leaves or boring into stems or fruits (Elmore 1943, Hayden et al. 2013). In addition to tomato, the larvae of *K.lycopersicella* feed on other solanaceous plants, such as potato or eggplant (Elmore 1943, Hayden et al. 2013). Although the hosts of some species of *Keiferia* are unknown (Povolný 1990, King and Montesinos 2012), all plants recorded so far as feeding substrate for these micromoths are members of the family Solanaceae (Powell and Povolný 2001, Povolný 2004, Hayden et al. 2013).

In the course of searches for lepidopteran larvae associated with plants native to the Atacama Desert in northern Chile, Gnorimoschemini micromoths were reared from leaf miner larvae collected on the native shrub *Trixiscacalioides* (Kunth) D. Don (Asteraceae). Subsequent examination revealed that the obtained micromoths belong to an undescribed species whose genitalia morphology fits that of *Keiferia* (Busck 1939, Povolný 1967). Since all the previous host records for members of this genus involve solanaceous plants (Powell and Povolný 2001, Povolný 2004, Hayden et al. 2013), the generic assignment of the new species was assessed by phylogenetic analysis of mitochondrial DNA sequences, a procedure that has been successfully used in Gelechiidae (Nazari 2017, Metz et al. 2019, Corley et al. 2020).

The aim of this contribution is to provide a description of a new species of *Keiferia* from the Atacama Desert associated with *T.cacalioides*, a discovery that represents the first record of a member of the family Asteraceae as a host plant for this micromoth genus.

## Materials and methods

The adult specimens examined in this study were reared from leaf miner larvae collected on *T.cacalioides* in two localities of the Atacama Desert, the Azapa Valley (18°35'20"S, 69°49'29"W) and Cuesta El Águila (18°29'08"S, 69°51'55"W), at about 1200 and 1900 m elevation, respectively, in the Arica Province, northern Chile. The abdomen of each adult was removed and placed in hot potassium hydroxide (KOH) 10% for a few minutes for dissection of the genitalia, which were stained with Eosin Y and Chlorazol Black and mounted on slides with Euparal. Photos were taken with an iPhone 11 camera attached to a Leica M125 stereomicroscope and a Leica MC170 HD camera attached to a Leica DM1000 LED microscope. Morphological terminology for the genitalia follows Huemer and Karsholt (2010). The holotype, paratypes and their genitalia slides are deposited in the “Colección Entomológica de la Universidad de Tarapacá” (IDEA), Arica, Chile.

Genomic DNA was extracted from two legs of the holotype using the QIAamp Fast DNA Tissue Kit, following the manufacturer’s instructions. DNA purification, PCR amplification and sequencing of the barcode region (Hebert et al. 2003) with the primers LCO1490 and HCO2198 (Folmer et al. 1994) were performed at Macrogen Inc. (Seoul, South Korea). The PCR programme included 5 min at 94°C, 35 cycles of 30 s at 94°C, 30 s at 47°C, 1 min at 72°C and a final elongation step of 10 min at 72°C. The obtained sequence was deposited in the Barcode of Life Data System (BOLD) (Ratnasingham and Hebert 2007) under process ID NCMIC015-24. Additional sequences (one per species) were downloaded from this database to perform a Maximum Likelihood (ML) analysis. The alignment included members of *Keiferia* with species-level identification, the type species of other Gnorimoschemini genera represented in the New World and members of *Stegasta* Meyrick, 1904, a genus belonging to Gelechiini, which is the sister group of Gnorimoschemini (Karsholt et al. 2013). The software MEGA11 (Tamura et al. 2021) was used to perform sequence alignment with the ClustalW method and to estimate sequence divergence with the Kimura 2-Parameter (K2P) method (Kimura 1980). The substitution saturation of the alignment was assessed with the Xia test in the software DAMBE7 (Xia 2018). The ML analysis was performed using the software IQTREE 1.6.12 (Nguyen et al. 2014) in the web interface W-IQ-TREE (Trifinopoulos et al. 2016) with data partitioned to codon position. ModelFinder (Kalyaanamoorthy et al. 2017) selected TN+F+G4, TN+F+I and HKY+F+G4 as the best fit models for 1^st^, 2^nd^ and 3^rd^ partitions, respectively. Branch support was assessed with 10000 replications of the Shimodaira-Hasegawa-like approximate likelihood ratio test (SH-aLRT) (Guindon et al. 2010) and ultrafast bootstrap (UFBoot) (Hoang et al. 2017). The unrooted tree was visualised in FigTree (Rambaut 2014) to root on *Stegasta*.

## Taxon treatments

### Keiferia azapaensis sp. nov.

84304A5E-D32D-5A7E-A2AD-6DA633EA8B81

BD5B684A-2B38-4799-90B7-6491F19868E5

#### Materials

- **Type status:** Holotype. **Occurrence:** catalogNumber: IDEA-LEPI-2024-25; sex: male; lifeStage: adult; preparations: Genitalia slide HAV1837; associatedSequences: BOLD Process ID: NCMIC015-24; occurrenceID: 0D8FBEFA-5B0D-5114-9712-32A1F039D8F6; **Location:** country: Chile; stateProvince: Arica; locality: Azapa Valley, 42 km; verbatimElevation: 1200 m; verbatimCoordinates: 18°35'20"S 69°49'29"W; **Identification:** identifiedBy: Héctor A. Vargas; **Event:** samplingProtocol: Male adult reared from leaf miner larva on Trixiscacalioides; verbatimEventDate: May 2021; **Record Level:** type: PhysicalObject; institutionCode: Colección Entomológica de la Universidad de Tarapacá (IDEA); basisOfRecord: PreservedSpecimen
- **Type status:** Paratype. **Occurrence:** catalogNumber: IDEA-LEPI-2024-28; sex: male; lifeStage: adult; preparations: Genitalia slide HAV1834; occurrenceID: EBFC653B-4CAB-55EA-B8A4-A890252D9844; **Location:** country: Chile; stateProvince: Arica; locality: Azapa Valley, 42 km; verbatimElevation: 1200 m; verbatimCoordinates: 18°35'20"S 69°49'29"W; **Identification:** identifiedBy: Héctor A. Vargas; **Event:** samplingProtocol: Male adult reared from leaf miner larva on Trixiscacalioides; verbatimEventDate: May 2017; **Record Level:** type: PhysicalObject; institutionCode: Colección Entomológica de la Universidad de Tarapacá (IDEA); basisOfRecord: PreservedSpecimen
- **Type status:** Paratype. **Occurrence:** catalogNumber: IDEA-LEPI-2024-30; sex: male; lifeStage: adult; preparations: Genitalia slide HAV1836; occurrenceID: FD869A96-5332-59E3-B967-A440604AC07B; **Location:** country: Chile; stateProvince: Arica; locality: Azapa Valley, 42 km; verbatimElevation: 1200 m; verbatimCoordinates: 18°35'20"S 69°49'29"W; **Identification:** identifiedBy: Héctor A. Vargas; **Event:** samplingProtocol: Male adult reared from leaf miner larva on Trixiscacalioides; verbatimEventDate: May 2017; **Record Level:** type: PhysicalObject; institutionCode: Colección Entomológica de la Universidad de Tarapacá (IDEA); basisOfRecord: PreservedSpecimen
- **Type status:** Paratype. **Occurrence:** catalogNumber: IDEA-LEPI-2024-31; sex: male; lifeStage: adult; preparations: Genitalia slide HAV1838; occurrenceID: 8AC21E20-7171-55B4-A08F-A379496BB167; **Location:** country: Chile; stateProvince: Arica; locality: Azapa Valley, 42 km; verbatimElevation: 1200 m; verbatimCoordinates: 18°35'20"S 69°49'29"W; **Identification:** identifiedBy: Héctor A. Vargas; **Event:** samplingProtocol: Male adult reared from leaf miner larva on Trixiscacalioides; verbatimEventDate: May 2017; **Record Level:** type: PhysicalObject; institutionCode: Colección Entomológica de la Universidad de Tarapacá (IDEA); basisOfRecord: PreservedSpecimen
- **Type status:** Paratype. **Occurrence:** catalogNumber: IDEA-LEPI-2024-29; sex: female; lifeStage: adult; preparations: Genitalia slide HAV1835; occurrenceID: 5967C16B-C91B-5984-BE5F-136677D2A1C4; **Location:** country: Chile; stateProvince: Arica; locality: Azapa Valley, 42 km; verbatimElevation: 1200 m; verbatimCoordinates: 18°35'20"S 69°49'29"W; **Identification:** identifiedBy: Héctor A. Vargas; **Event:** samplingProtocol: Female adult reared from leaf miner larva on Trixiscacalioides; verbatimEventDate: May 2017; **Record Level:** type: PhysicalObject; institutionCode: Colección Entomológica de la Universidad de Tarapacá (IDEA); basisOfRecord: PreservedSpecimen
- **Type status:** Paratype. **Occurrence:** catalogNumber: IDEA-LEPI-2024-26; sex: female; lifeStage: adult; preparations: Genitalia slide HAV1597; occurrenceID: 69D82B25-258E-50B5-9BAC-BFE14BEFCCCD; **Location:** country: Chile; stateProvince: Arica; locality: Cuesta El Ágila; verbatimElevation: 1900 m; verbatimCoordinates: 18°29'08"S 69°51'55"W; **Identification:** identifiedBy: Héctor A. Vargas; **Event:** samplingProtocol: Female adult reared from leaf miner larva on Trixiscacalioides; verbatimEventDate: March 2011; **Record Level:** type: PhysicalObject; institutionCode: Colección Entomológica de la Universidad de Tarapacá (IDEA); basisOfRecord: PreservedSpecimen
- **Type status:** Paratype. **Occurrence:** catalogNumber: IDEA-LEPI-2024-27; sex: male; lifeStage: adult; preparations: Genitalia slide HAV1598; occurrenceID: 50C1674C-1482-5BC1-9507-F3A041F9372B; **Location:** country: Chile; stateProvince: Arica; locality: Cuesta El Ágila; verbatimElevation: 1900 m; verbatimCoordinates: 18°29'08"S 69°51'55"W; **Identification:** identifiedBy: Héctor A. Vargas; **Event:** samplingProtocol: Male adult reared from leaf miner larva on Trixiscacalioides; verbatimEventDate: March 2011; **Record Level:** type: PhysicalObject; institutionCode: Colección Entomológica de la Universidad de Tarapacá (IDEA); basisOfRecord: PreservedSpecimen

#### Description

**Figure 1.**
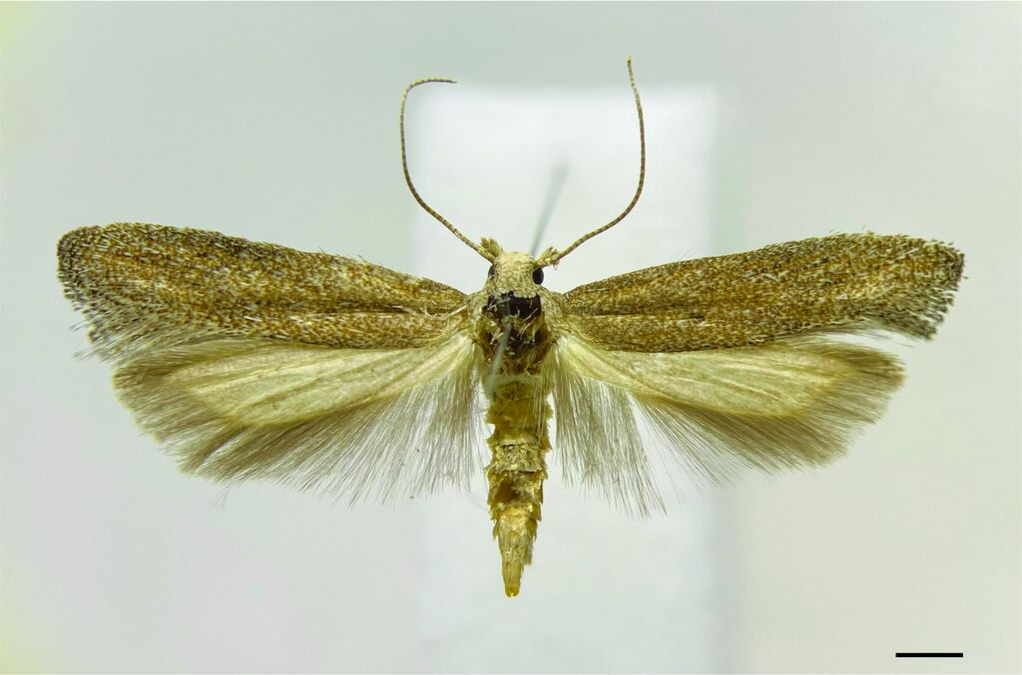
Holotype male of *Keiferiaazapaensis* sp. nov., dorsal view. Scale bar 1 mm.

**Figure 2.**
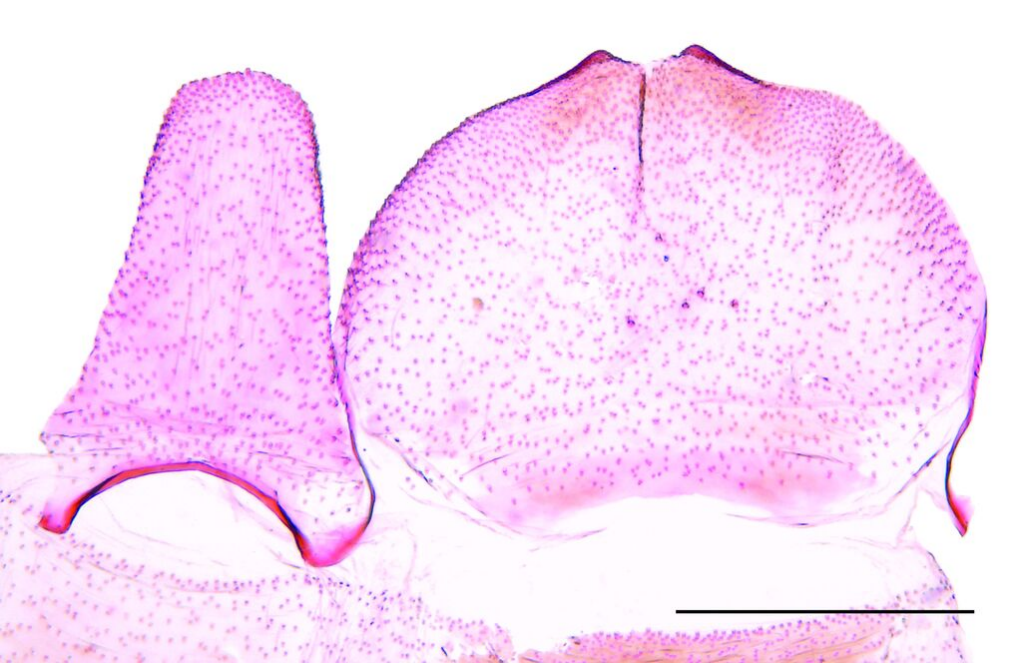
Male abdominal segment VIII of *Keiferiaazapaensis* sp. nov.; tergum on the left; sternum on the right. Scale bar 0.5 mm.

**Figure 3.**
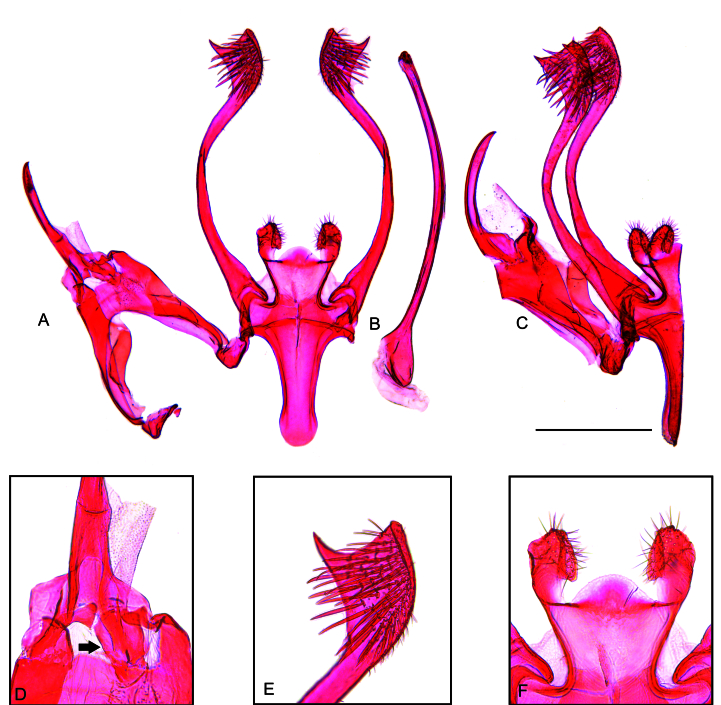
Male genitalia of *Keiferiaazapaensis* sp. nov. **A** unrolled, phallus removed; **B** phallus, lateral view; **C** lateral view, phallus removed; **D** gnathos (black arrow); **E** inner surface of the tip of the right valva; **F** processes of the vinculum. Scale bar 0.5 mm.

**Male** (Fig. 1). **Head**: Frons and vertex whitish-brown; maxillary palp whitish-brown; labial palp mostly whitish-brown with scattered dark brown scales; haustellum with whitish-brown scales. Antenna greyish-brown, filiform, length about two-thirds the forewing. **Thorax**: Greyish-brown dorsally, whitish-brown laterally; legs mostly greyish-brown with scattered whitish-brown scales. **Forewing**: Greyish-brown with scattered dark brown scales mostly on distal half; fringe similar in colour; length 6.1–6.2 mm. **Hindwing**: Whitish-brown with greyish-brown fringe. **Abdomen**: Mostly whitish-brown with terga I-III yellowish-brown. Tergum VIII (Fig. 2) tongue-like, elongated, progressively narrowing backwards, length in the middle about 1.1 times maximum width; anterior margin broadly concave, strongly sclerotised; posterior margin rounded. Sternum VIII (Fig. 2) elliptical, broad, length similar to tergum VIII, maximum length about 0.7 times the maximum width; posterior margin with two small rounded projections near the middle. **Male genitalia** (Fig. 3): Tegumen narrow; anterior margin smooth; posterior margin slightly sinuous. Uncus large, pointed, hook-shaped; length similar to tegument height. Gnathos with a small central plate. Vinculum with a trapezium-shaped posterior projection in the middle; vincular processes with slightly expanded, setose posterior half, separated by a semicircular plate. Saccus similar in length to uncus, flattened, separated from vinculum by a nearly straight transverse line, lateral margins slightly concave, tip rounded. Valva about twice the length of the saccus, narrow, sinuous, with a pointed subapical dorsal expansion with setose inner side. Phallus narrow, elongated, curved, length about 1.2 times the valva, with small terminal sclerite; coecum slightly swollen.

**Figure 4.**
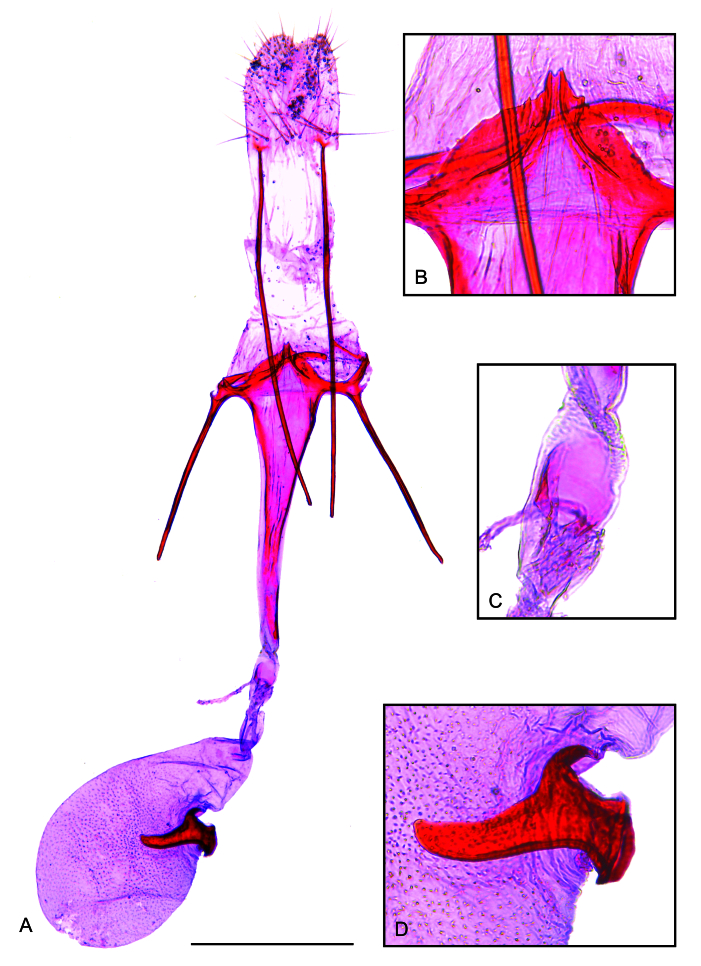
Female genitalia of *Keiferiaazapaensis* sp. nov. **A** ventral view; **B** posterior tip of the antrum; **C** colliculum; **D** signum. Scale bar 0.5 mm.

**Female**. Similar to male in wing pattern and size. **Female genitalia** (Fig. 4): Papillae anales broadly rounded posteriorly, with a few scattered setae. Posterior apophyses rod-shaped, length about three times the papillae anales. Anterior apophyses rod-shaped, length about half the posterior apophyses. Tergum VIII as a fine stripe ventrally continuous with the posterior tip of the anterior apophyses and outer tip of the sternum VIII. Sternum VIII as a short fine stripe with inner tip continuous with the antrum. Ostium bursae at the posterior end of the antrum. Antrum flattened, progressively narrowing forwards, length about 1.5 times the anterior apophyses; ventral part projecting posteriorly as a semicircular plate with margin serrated in the middle and two divergent curved lines arising from the two central teeth. Ductus bursae mainly membranous, about one-third the length of the antrum; a small colliculum close to anterior tip of the antrum; inception of ductus seminalis near the anterior margin of the colliculum. Corpus bursae pear-shaped, mostly membranous, length similar to the antrum, with a stout, slightly curved signum with rounded tip.

#### Diagnosis

*Keiferiaazapaensis* sp. nov. is recognised by the trapezium-shaped posterior projection of the vinculum, the vincular processes separated by a semicircular plate and the valva with a pointed subapical dorsal expansion in the male genitalia and the antrum with a semicircular posterior projection with margin serrated in the middle and the stout, slightly curved signum with rounded tip in the female genitalia. Although the male genitalia of *K.azapaensis* sp. nov. resemble those of *K.lobata* Povolný, 1990 from Bolivia (Povolný 1990; fig. 71), the latter lacks a semicircular plate separating the vincular processes and has a prominent straight terminal spine on the valva. Furthermore, the uniformly sclerotised vincular processes of *K.azapaensis* sp. nov. contrast with those of *K.lobata*, which have strongly sclerotised margins. The female of *K.lobata* remains unknown, impeding comparisons. However, the two species also differ in forewing pattern, as the forewing of *K.azapaensis* sp. nov. is greyish-brown with scattered dark brown scales mostly on the distal half, while that of *K.lobata* is ferruginous with whitish admixture basally, followed by a striking black spot before the middle and a mixture of ferruginous, whitish and black on the distal half and black scales in tornal and apical areas (Povolný 1990).

#### Etymology

The specific epithet derives from the type locality.

#### Distribution

**Figure 5.**
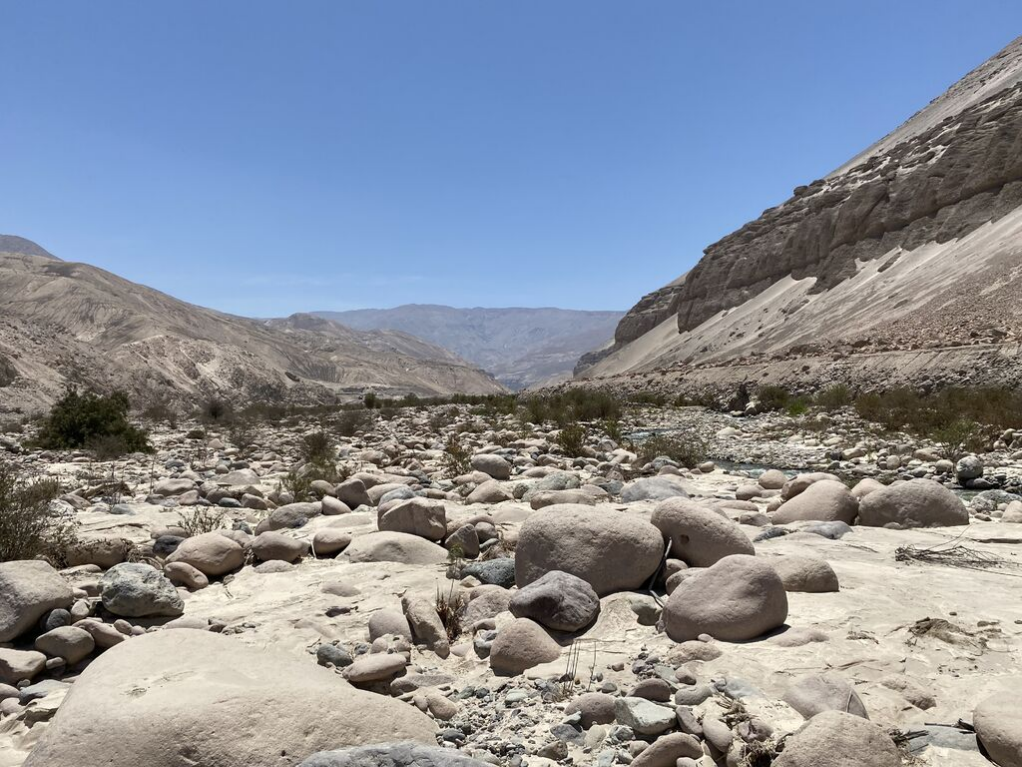
Type locality of *Keiferiaazapaensis* sp. nov. in the Azapa Valley, Atacama Desert, northern Chile.

*Keiferiaazapaensis* sp. nov. has been recorded only in two localities of the Atacama Desert, the Azapa Valley (Fig. 5) and Cuesta el Águila, between about 1200 and 1900 m elevation in the Arica Province of northern Chile.

#### Host plant

**Figure 6.**
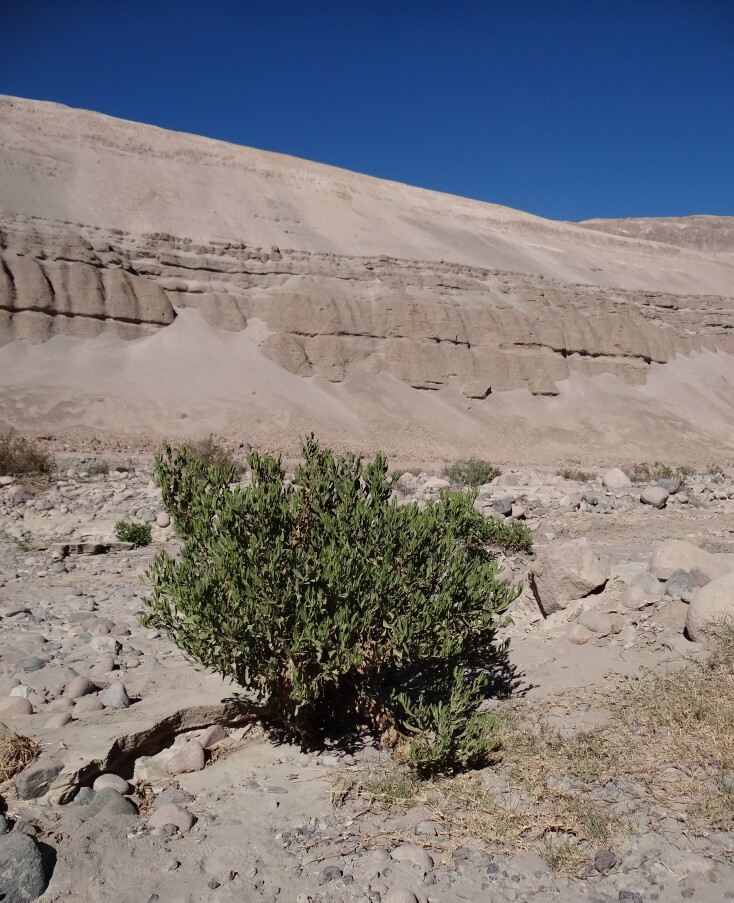
*Trixiscacalioides* (Kunth) D. Don, the host plant of *Keiferiaazapaensis* sp. nov., growing at the Azapa Valley, Atacama Desert, northern Chile.

*Trixiscacalioides* (Fig. 6) is the only host plant documented for *K.azapaensis* sp. nov. This shrub also occurs in Argentina, Bolivia, Ecuador, Paraguay and Peru (Katinas 1996, GBIF 2023); its Chilean range is restricted to the Arica y Parinacota and Tarapacá Regions, in the northernmost part of the country (Moreira-Muñoz et al. 2012).

#### DNA barcoding

**Figure 7.**
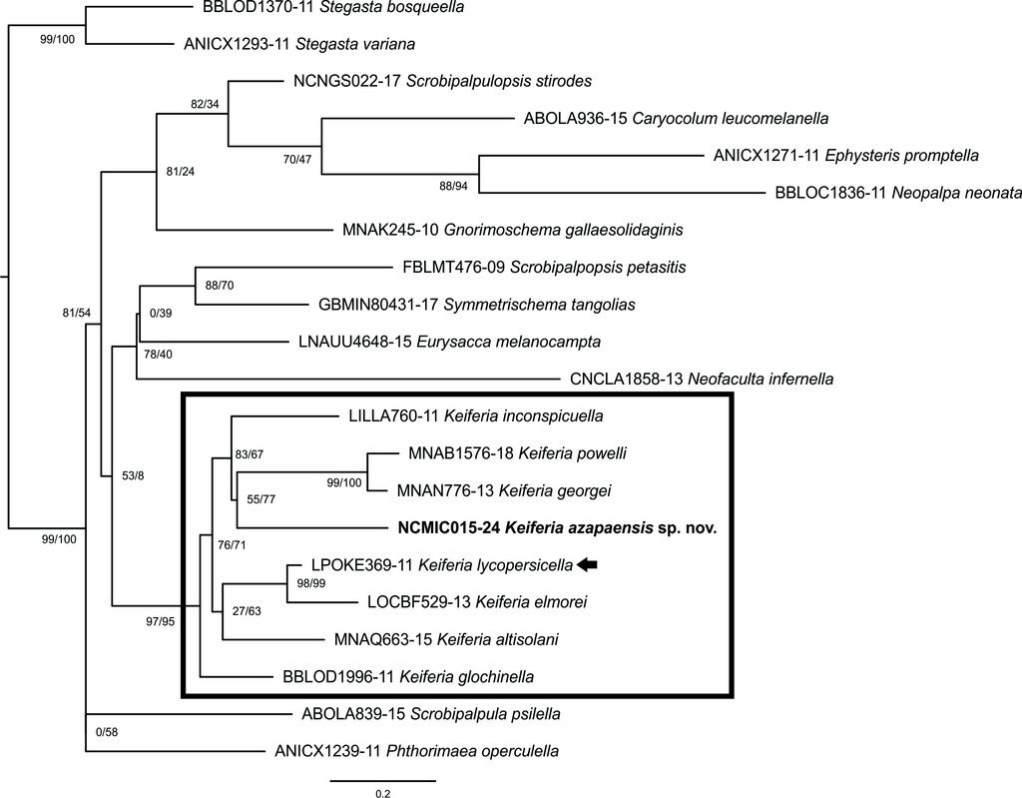
Maximum Likelihood tree of *Keiferiaazapaensis* sp. nov. (bold) and representatives of Gnorimoschemini, based on mitochondrial DNA sequences. The tree was rooted on *Stegasta* Meyrick, 1904, a member of Gelechiini, sister group of Gnorimoschemini. BOLD Process ID to the left of each species; rectangle delimits *Keiferia* Busck, 1939; arrow indicates its type species; numbers represent SH-aLRT/UFBoot values (10000 replicates).

Genetic divergence between the new species and other members of *Keiferia* ranged from 9.3% (K2P), with *K.glochinella* (Zeller, 1873) and *K.lycopersicella*, to 11.2% (K2P), with *K.elmorei* (Keifer, 1936) and *K.powelli* Povolný, 2004. Genetic divergence between other members of *Keiferia* ranged from 3.1%, between *K.georgei* (Hodges, 1986) and *K.powelli* Povolný, 2004, to 9.4%, between *K.inconspicuella* (Murtfeldt, 1883) and *K.powelli*. The alignment was suitable for phylogenetic analysis, as no evidence of stop codons was detected and the Xia test found an index of substitution saturation smaller than the critical value (ISS < ISS.C; p < 0.001). The ML analysis (Fig. 7) placed all the members of *Keiferia* into a well-supported cluster (97% SH-aLRT, 95% UFBoot), including the new species. However, the relationships of the latter with other representatives of the genus were poorly resolved.

## Discussion

Amongst the New World Gnorimoschemini, members of *Keiferia* are clearly recognised, based on the well-developed uncus, weak gnathos and apically enlarged valva of the male genitalia (Busck 1939, Povolný 1967, Powell and Povolný 2001). In accordance with morphology, the ML analysis provided high support for the monophyly of *Keiferia*. However, the relationships amongst the species of this genus were poorly resolved, with the exceptions of *K.elmorei* + *K.lycopersicella*, supported by 98% SH-aLRT and 99% UFBoot and *K.georgei* (Hodges, 1985) + *K.powelli*, supported by 99% SH-aLRT and 100% UFBoot. Interestingly, these two well-supported species pairs are congruent with morphology, as the genitalia of *K.elmorei* are very similar to those of *K.lycopersicella* and those of *K.georgei* to those of *K.powelli* (Hodges 1985, Powell and Povolný 2001, Povolný 2004). Due to the remarkable resemblance in genitalia morphology, *K.elmorei* was synonymised with *K.lycopersicella* (Povolný 1967), but later was recognised as a distinct species, based on morphological and biological attributes (Powell and Povolný 2001).

Although the ML analysis placed *K.azapaensis* sp. nov. as sister to *K.georgei* + *K.powelli*, the support for this cluster was low (55% SH-aLRT, 77% UFBoot). However, the poorly-resolved relationships of the newly-discovered species to congenerics can be understood as a result of the limited taxon sampling, as the alignment included mostly Nearctic members of the genus and did not include *K.lobata*, the closest species based on morphology. Further molecular phylogenetic studies, based on wider taxon sampling and additional molecular markers, would be extremely useful to improve the understanding of the evolutionary relationships between *K.azapaensis* sp. nov. and other members of *Keiferia*.

As all previous host records for species of *Keiferia* involved exclusively solanaceous plants (Powell and Povolný 2001, Povolný 2004, Hayden et al. 2013), the discovery of the trophic association between *K.azapaensis* sp. nov. and *T.cacalioides* represents the first record of a member of Asteraceae as a host plant for this micromoth genus. Since host plants remain undocumented for some South American species of *Keiferia* (Povolný 1990, King and Montesinos 2012), further fieldwork is encouraged to fill this gap, as this basic information is fundamental to understand the host plant use patterns in an evolutionary framework.

## Supplementary Material

XML Treatment for Keiferia azapaensis
